# Development and Validation of a High Throughput System for Discovery of Antigens for Autoantibody Detection

**DOI:** 10.1371/journal.pone.0040759

**Published:** 2012-07-18

**Authors:** Isabel K. Macdonald, Jared Allen, Andrea Murray, Celine B. Parsy-Kowalska, Graham F. Healey, Caroline J. Chapman, Herbert F. Sewell, John F. R. Robertson

**Affiliations:** 1 Oncimmune Ltd, Nottingham City Hospital, Nottingham, United Kingdom; 2 Centre of Excellence for Autoimmunity in Cancer, University of Nottingham, Nottingham, United Kingdom; King’s College London, United Kingdom

## Abstract

An assay employing a panel of tumor-associated antigens has been validated and is available commercially (*Early*CDT®-Lung) to aid the early detection of lung cancer by measurement of serum autoantibodies. The high throughput (HTP) strategy described herein was pursued to identify new antigens to add to the *Early*CDT-Lung panel and to assist in the development of new panels for other cancers. Two ligation-independent cloning vectors were designed and synthesized, producing fusion proteins suitable for the autoantibody ELISA. We developed an abridged HTP version of the validated autoantibody ELISA, determining that results reflected the performance of the *Early*CDT assay, by comparing results on both formats. Once validated this HTP ELISA was utilized to screen multiple fusion proteins prepared on small-scale, by a HTP expression screen. We determined whether the assay performance for these HTP protein batches was an accurate reflection of the performance of R&D or commercial batches. A HTP discovery platform for the identification and optimal production of tumor- associated antigens which detects autoantibodies has been developed and validated. The most favorable conditions for the exposure of immunogenic epitopes were assessed to produce discriminatory proteins for use in a commercial ELISA. This process is rapid and cost-effective compared to standard cloning and screening technologies and enables rapid advancement in the field of autoantibody assay discovery. This approach will significantly reduce timescale and costs for developing similar panels of autoantibody assays for the detection of other cancer types with the ultimate aim of improved overall survival due to early diagnosis and treatment.

## Introduction

The precise role of the immune system in cancer remains to be fully elucidated despite decades of research. It is known that a patient may display an immune response to tumor cells or tumor-derived or associated antigens (TAAs). This can result in the production of autoantibodies (AAb) against antigenic constituents of the cancer patient’s own tissues. Although the implications of this for tumor progression remain unclear, the presence of AAb in patients with cancer has raised the possibility that their measurement may be used as an aid to early cancer detection as well as selection and monitoring of treatment [1]–[6].

Improved cancer research reagents for use in novel profiling and screening strategies are being sought in order to speed up our understanding of the immune system in cancer and our ability to harness it for improving healthcare [7]. If such strategies are to be accurate and meaningful, they need to be reproducible with high sensitivity and specificity, incorporating rigorous quality control. They must undergo quality assurance during development and in subsequent laboratory and clinical use. One potential problem with new technologies is that there may be no accepted standard against which to validate them. The *Early*CDT-Lung test, which measures AAb against a panel of TAAs, is currently used to aid early detection of lung cancer. This test has been approved by Clinical Laboratory Improvement Amendments (CLIA) and as such meets the criteria of an accepted standard, having itself been through both technical and clinical validation [6], [8], [9].

*Early*CDT-Lung took three years from pilot studies [5] resulting in a decision to pursue a lung cancer test, to publication of the technical and clinical validation studies [6], [8]. This panel was developed by screening many TAAs using a 5 point assay of recombinant protein batches (from *E.Coli*) and testing seven per plate. Panel antigens were selected by assessing their suitability to detect and bind serum AAb and their ability to differentiate between cancer patients and normal individuals. Externally produced batches of promising leads were then tested for final assessment of their suitability for inclusion in the commercially available *Early*CDT-Lung test. While this discovery and development method was successful, as the *Early*CDT-Lung panel is testament to, it was also a lengthy and costly process. The current seven TAA *Early*CDT-Lung test has a reported specificity of 93% and a sensitivity of 41% for the detection of lung cancer [10]. To improve these performance characteristics additional AAb may need to be added to the panel.

AAb tests for other cancer types (e.g. breast, colon, liver, and ovary) will now follow but in order to speed up the development timelines, a more efficient and high throughput (HTP) method of lead discovery was required. One major limiting factor to most screening strategy developments is acquiring an appropriate volume of sample, from a large number of suitable individuals, in order to both test and validate the assay during development. Acquiring suitable cohorts of patient serum is a lengthy process and in many instances only limited volumes can be obtained. A HTP screening strategy, which not only allows a more rapid and cost efficient method to identify lead TAAs from the vast amount of biomarkers regularly appearing in the literature, but also uses serum economically, would provide a significant step towards reducing research and development (R&D) costs and timelines.

Developments in the fields of HTP cloning, and protein expression and purification, mainly by crystallography/structural genomic consortiums, have been on-going for over a decade and regularly utilize multi-well plate formats [11]. One such development is ligation independent cloning (LIC), which does not require the use of restriction enzymes or ligases and instead utilizes the exo-nuclease activity of T4 DNA polymerase to prepare a linear vector with long single 5′ stranded ends which anneal to similarly prepared complementary strands of the PCR amplified gene of interest [12]. When introduced into a suitable host, the host’s native enzymes ligate and propagate the plasmid. These properties combined with the higher efficiencies of LIC over more traditional methods, make this approach amenable to preparation of 96 constructs in parallel in less than two weeks at greater than 80% efficiency, utilizing microtiter plates and commercially available HTP DNA purification and plasmid preparation methods.

We therefore describe here the development and validation of our own LIC strategy to suit our specific needs: primarily a vector to produce bacterially expressed fusion proteins of TAAs, suitable for our assay and purification protocols. The expression and purification of the resultant TAA fusion proteins were then optimized by developing HTP strategies. These strategies exploited established methodologies [13] to produce mg quantities of up to 48 proteins in parallel in a matter of weeks; again utilizing multi-well plate technologies. The resultant proteins were subsequently investigated for their suitability for inclusion in a panel of TAAs to select and detect AAb by a 2 point serological ELISA assay with the ultimate aim being the production of new and improved early cancer detection tests. The HTP strategy described was validated as a fast, reliable and cost effective method for identification of new antigens for inclusion in AAb panels for early detection of cancer.

## Materials and Methods

### Ethics Statement

Samples used in this study were either collected within the East Midlands area of the UK with participant full written informed consent and approval from the University of Nottingham Medical Research Ethics Committee (Ethics Reference Number BT/07/2007) or purchased from Kiev Biopharma who obtained full written informed consent at the enrolment sites.

### LIC Cloning

**Figure 1:**
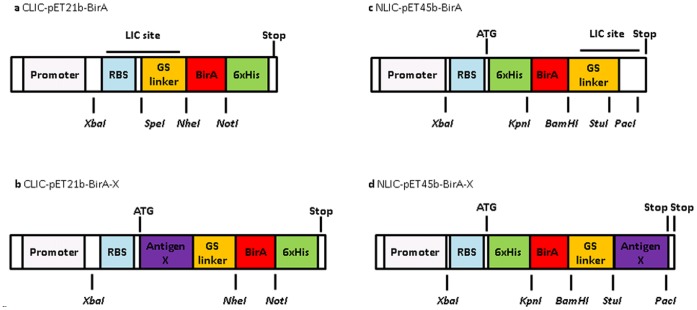
LIC-compatible expression vectors. The LIC sites were designed to incorporate a restriction site flanked by LIC compatible annealing sites.

LIC compatible *E.Coli* protein expression vectors were generated using the commercial vectors pET21b and pET45b (Novagen, Merck) (Figure 1). The BirA tag [14]–[16] is a 129 amino acid biotin purification tag used in Pinpoint Xa1-3 vectors (Promega) and was used as an assay control tag. This was introduced by PCR amplification and cloned into both commercial vectors. The LIC restriction and annealing sites were appropriately designed [12], and the required short DNA sequences were synthesized (Sigma) and introduced by restriction site cloning into the BirA vectors (Figure 1).

For the CLIC vector the *SpeI* restriction site and the downstream LIC compatible site were incorporated into pET21b-BirA by designing two short DNA sequences (Sigma) as if already digested, and after denaturing and annealing they were cloned by restriction and ligation into the *NdeI* and *NheI* sites of the pET21b C-BirA vector. An LIC incompatible sense G residue (part of the *NdeI* site) was deleted by site directed mutagenesis, resulting in the expression vector CLIC-pET21b-BirA encoding for C-terminal-6xHis-BirA fusion proteins after LIC of antigen-X open reading frames into the LIC site. For the NLIC vector the *StuI* restriction site and an upstream LIC compatible site were incorporated into pET45b-BirA by designing two DNA sequences as if already digested. The NLIC expression vector, NLIC-pET45b-BirA, encodes N-terminal-6xHis-BirA-Antigen-X fusion proteins after LIC of Antigen-X open reading frames into the LIC site. Start and stop codons were provided by the vector; another stop codon immediately downstream of the target open reading frame was added by the reverse primer during PCR amplification (Figure 1). The prepared vectors were denoted XLIC where X denotes the position of the DNA encoding the purification and assay tags (a 6x histidine purification tag and a BirA assay control tag) at the N or C termini of the final translated fusion protein. Both vectors were ampicillin resistant and selected throughout using carbenicillin.

### HTP Cloning (HTPC)

LIC vectors were linearized by incubation with the appropriate restriction enzyme (*StuI* for NLIC; *SpeI* for CLIC; New England Biologicals (NEB)), followed by heat inactivation. The CLIC vector was further incubated with mung bean phosphatase to prepare blunt ends, followed by enzyme inactivation (0.1% SDS). Linear blunt ended vector was purified from un-digested circular vector by agarose gel electrophoresis and gel-extracted (QIAquick gel extraction kit, QIAGEN). The long 5′ LIC compatible overhangs were generated by incubating the pure DNA with T4 DNA polymerase (Novagen, Merck) in the presence of dCTP and dithiothreitol (DTT). The reaction mixture was then heat inactivated and stored at −20°C.

The DNA encoding the human TAA proteins were amplified by PCR (KOD Hot Start Master Mix; Novagen), using IMAGE clone templates (Geneservice) and appropriate primers. Template DNA was removed from PCR products by *DpnI* digestion (NEB) and reactions were purified (AMPure XP, Agencourt) and analyzed by agarose gel electrophoresis. The LIC ready PCR products were prepared by T4 DNA polymerase (as above), substituting dGTP in place of dCTP.

The vector and inserts were designed so that the two conversely T4 treated fragments (>12 nucleotide 5′ overhangs) would anneal when combined, and could then be introduced into a suitable host where the host’s native enzymes ligate then propagate the plasmid. The LIC ready vector and purified PCR products were annealed, EDTA was added and after a further incubation reactions were stored at −20°C.

LIC reactions and a negative control (LIC ready vector only) were transformed into *E.Coli* (NovaBlue giga, Novagen) and transformation cultures were grown on Luria Bertani (LB) agar. To estimate cloning efficiencies multiple colonies were picked for each LIC construct and plasmid DNA was prepared (CosMC kit, Agencourt). Insert specific PCR (using an insert specific cloning primer and a T7 promoter or terminator primer) was performed on both clones and the product was analyzed for an insert of the correct size by agarose gel electrophoresis. Construct identification was verified by DNA sequencing (Source Bioscience).

### HTP Expression Screen (HTPE)

Our final optimized standard expression screen was deduced from expression of well over 50 TAA constructs, LIC and non LIC vector constructs. A large variety of plates, plate seals, host cells, media, expression media volumes, incubation temperatures, shaking speeds, induction conditions/methods, post induction incubation periods and various supplements were investigated. Multiple plates of the same expression were set up depending on the number of serum samples to be tested by the HTP ELISA. For the resultant screen the expression constructs were transformed into *E. coli* strain BL21 (DE3) RIPL (Agilent) and transformants were grown on chloramphenicol supplemented LB agar. Colonies were picked and incubated (37°C, 200 rpm) overnight in sterile LB media supplemented with glucose. The constructs were over-expressed by inoculation of enriched media with the LB cultures. Culture plates were incubated (37°C, 200 rpm) until OD at 600 nm were greater than 0.4OD Units. Over-expression was induced (auto-induction or IPTG) and the expression cultures were incubated overnight (200 rpm, 25 or 37°C).

### Lysis & Purification

Cultures were harvested by centrifugation (2100×g, 10 minutes) and the pellets were stored at −80°C for at least 40 minutes to aid cell lysis. Cell pellets were further lyzed by incubation (400 rpm, 20 minutes) with protease inhibitor cocktail V, (Calbiochem) and BugBuster® (Novagen). The plates were centrifuged again and the cleared soluble lysates were aspirated to a new plate. The pellets were further incubated with BugBuster and the lysates were aspirated and stored at 4°C until purification. The remaining insoluble inclusion body (IB) pellets were solubilized (guanidine hydrochloride) by vortexing and shaker incubation (400 rpm, 1 hour). The IB lysates were cleared as before, aspirated and stored at 4°C until purification.

Soluble and insoluble lysates were purified using HIS-Select filter plates (Sigma) according to the manufacturer’s instructions. Briefly, lysates were loaded onto the pre-equilibrated HIS-select plate (up to 2 mg of protein can be purified per well). Each well was washed (guanidine followed by urea buffer, containing 5 mM and 10 mM imidazole, respectively) and constructs were then refolded prior to elution by washing and incubating with refold (glutathione redox buffer) and storage buffers (glycerol and TRIS buffer) prior to elution (storage buffer containing 250 mM imidazole). All purified protein samples had aliquots removed for SDS-PAGE analysis prior to storage at −20°C.

### Characterization and Quantification

The reduced protein samples were analyzed for purity, yield and molecular weight by SDS-PAGE (4–20%, 17 wells, Expedeon) and run against a known molecular weight marker (Novex® Sharp unstained marker, Life Technologies) and bovine serum albumin (BSA) standards. Proteins were visualized (InstantBlue, Expedeon) and concentrations were estimated by comparison to BSA.

### Patient Samples

Collection and preparation of all serum samples has been described previously [8]. Cancer and control samples were all processed identically. Two cohorts of patients with newly diagnosed lung cancer were identified. Cohort 1 contained 32 lung cancer patients (median age 67; range 40–77) and 16 normal healthy control individuals (63.5; 40–75) while cohort 2 had 200 lung cancer patients (62; 34–87) and 200 healthy control samples (61.5; 34–87) respectively. Patients with lung cancer were individually matched for cohort 2, and half of cohort 1 patients, by age, gender and smoking history to a control individual with no previous history of malignant disease. In patients with lung cancer, blood samples were obtained after diagnosis but before receiving any anti-cancer treatment. Samples were either collected within the East Midlands area of the UK or purchased from Kiev Biopharma with full written informed consent being obtained at both sites.

### HTP ELISA (HTPA)

The abridged HTP assay was based upon the *Early*CDT indirect ELISA described previously [6], [8]; a brief description and any differences from the published assay are described herein. Purified recombinant antigens (21 per assay) were diluted to 100 nM and 50 nM. A tag control antigen (BirA) was included to allow subtraction of the signal due to nonspecific binding to bacterial contaminants (as in the *Early*CDT). A cancer control antigen was also included to allow inter-HTPA validation. The antigen chosen was a TAA with good cancer-normal differentiation. For data presented here, NY-ESO-1 BirA was employed.

A control plate where an anti-His tag monoclonal primary antibody (Novagen) was used in place of serum was included to validate plate coating success. SDS-PAGE analysis of TAA plate coating solutions was also carried out to verify plate layouts and protein dilutions.

The mean OD signals of the replicates of antigen coated wells were corrected by subtracting the mean signal of the tag control. Negative values were set to zero and any outliers were removed based on high intra-duplicate coefficients of variation (CV). Samples were judged to be positive for the presence of a specific AAb when the ELISA OD mean was above a cut-off for the corresponding antigen. Cut-offs were chosen for each antigen so that sensitivity was similar to that for the *Early*CDT assay (see below). For positivity assessment, samples were rejected as invalid for a particular antigen if both concentrations were rejected due to high CVs. If only one concentration was rejected, then the sample was rejected as invalid if the other concentration was negative, but was deemed positive if the other concentration was positive.

### R&D ELISA

The validated AAb assay, employing a 5 point titration of each individual antigen (R&D or commercial batch), was performed as previously described [6], [8] but without calibration. Data were tag corrected, negative values set to zero, outliers removed and OD cut-offs estimated as outlined above for HTPA.

### R&D *Early*CDT ELISA

This was performed as described previously [6], [8] using the commercial panel of antigens and applying calibration but without the full automated LIMS data cleaning used for the commercial test. The commercial RU cut-offs were used, so this assay represented the R&D version of the commercial *Early*CDT-Lung panel and as such provides a statistically robust correlation of how a sample would perform if sent for the commercial test.

### Comparison of Assays

Table 1 summarizes the differences between the three assay formats. Scatter plots where the ELISA OD means for each sample were plotted in groups according to assay format, antigen batch used, antigen concentration and sample disease class (cancer or normal) were constructed. Concordance between any two formats was calculated as the percentage of samples giving the same result (either positive or negative). For HTPA versus R&D *Early*CDT, the sensitivity had been equalized as stated above, so only the specificity needed to be compared.

**Table 1:** Summary of assay format characteristics.

Assay Format	HTPA	R&D Assay	EarlyCDT
**Antigen batches used**	HTPE, R&D or Commercial	R&D or Commercial	R&D or Commercial
**Antigen concentration**	50 & 100 nM	1.6, 5, 16, 50 & 160 nM	1.6, 5, 16, 50 & 160 nM
**Calibration**	None	None	Calibrated with specific pleural fluids
**Positivity**	OD signal > cut-off (50 or 100 nM)	OD signal > cut-off (50 or 160 nM)	RU dose response > antigen cut-off(50 or 160 nM
**Cut-off determination**	Estimated from mean +2 SD & scatterplots for cohort tested	Estimated from mean +2 SD & scatterplots for cohort tested	Monte Carlo optimized frommulti-centre cohorts

## Results

### HTPC

The two LIC vectors (Figure 1) were successfully synthesized and the LIC protocol was developed and optimized by preparing four constructs for each vector (N- and C-LIC vectors) achieving efficiencies of between 80 and 95%. The disparity in cloning efficiencies for the two vectors was considered to be due to vector purity: the number of colonies for the vector only control plate for CLIC and NLIC were 2 and 15 respectively, which would imply that the lower efficiency for NLIC was due to a larger amount of uncut circular vector remaining in the NLIC batch, a conclusion further supported by the presence of bands at 0.6 KBP in the failed clones (the size of empty NLIC vector i.e. no TAA sequence present) (data not shown). In subsequent HTP LIC reactions, where up to 96 clones have been prepared in parallel, preparation of two clones resulted in cloning efficiencies consistently over 80% (data not shown).

### HTPE Screen

**Figure 2:**
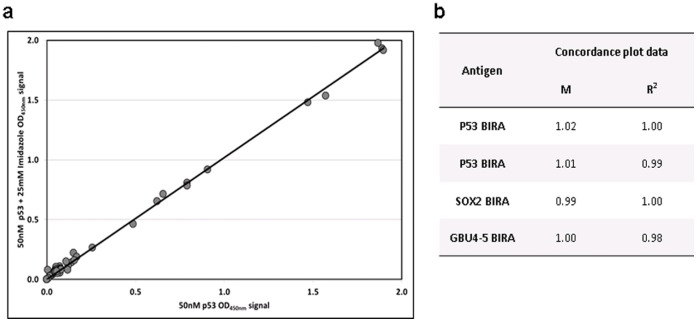
Imidazole concordance. Antigen solutions (50 nM) with and without imidazole (25 mM) were used to coat plates, and AAbs in cohort 1 serum samples were detected by ELISA (HTPA): a) an example of a concordance plot between the OD signal for p53 BirA with and without imidazole; b) the gradient (m) and fit (R^2^) values for the plot shown and the remaining cohort 1 data.

The HTPE recombinant proteins were expressed using an optimized standard screen deduced from extensive experiments. The final purification protocol was based upon current R&D batch purification procedures where possible [10], [13]. Purification of lysates was attempted using a variety of platforms (all nickel affinity based methods) with Sigma’s HIS-Select filter plate emerging as the preferred method, including refolding of the immobilized proteins on the HIS-Select resin. The resultant presence of imidazole had no effect on assay results (Figure 2) and so was not removed from HTPE batches prior to ELISA (it is removed for R&D and commercial batches).

### HTPA Antigen Concentrations

**Figure 3:**
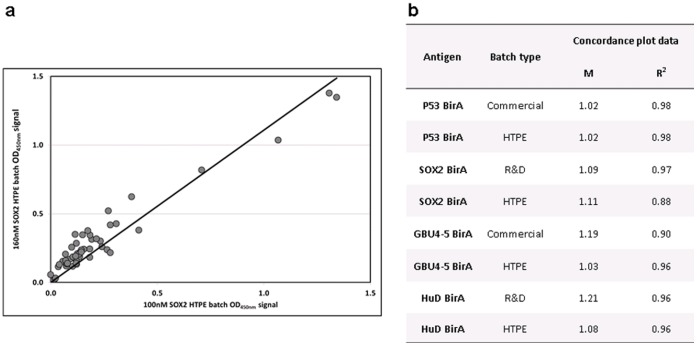
100 vs 160 nM concordance. HTPE, R&D and commercial batches of several antigens at 100 and 160 nM were used to coat plates and AAbs in cohort 1 serum samples were detected by HTPA: a) an example of a concordance plot between the 100 and 160 nM OD data for a HTPE batch of SOX2 BirA; b) the gradient (m) and fit (R2) values for the plot shown and the remaining cohort 1 data.

The two concentrations (50 & 100 nM) were determined as being the most appropriate for the HTPA following investigation of all five antigen concentration points (1.6, 5, 16, 50 & 160 nM) in the R&D assay for several antigens (data not shown). The two highest concentrations (50 & 160 nm) gave the strongest signal, but 100 nM concentration gave comparable and reproducible results to 160 nM (Figure 3) while providing optimal protein usage for HTPA i.e. more samples can be tested per HTPE batch.

**Figure 4:**
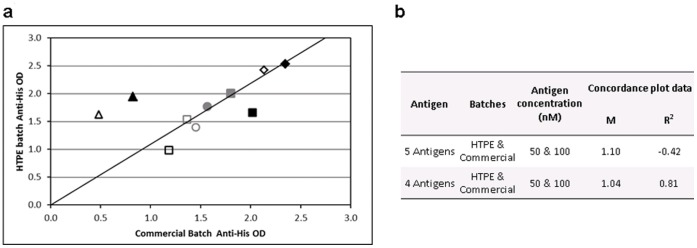
Anti-His Batch concordance. Antigen solutions of Commercial and HTPE batches at 50 nM (outline) and 100 nM (filled) were used to coat plates, and proteins were detected using anti-his antibody by ELISA (HTPA) for five antigens: CAGE BirA (grey circle), Annexin I BirA (grey square), Cathepsin D BirA (black square), LMYC2 (black diamond) and Mesothelin BirA (black triangle). a) Concordance plot between the OD signal for all antigens at both concentrations; b) the gradient (m) and fit (R^2^) values for the plot shown with and without Mesothelin. The concordance for this antigen was poor, demonstrated by the fact that when the values are taken out a much greater concordance is observed for the remaining four antigens.

A control plate to which anti-His tag MAb (Novagen) had been added in place of serum was included for every assay to confirm successful plate coating. The data were used during development to validate the HTPE quantification method by comparing the signal of HTPE to commercial batches at two concentrations (50 & 100 nM) for six antigens. Five out of the six antigens gave good concordance (Figure 4) indicating that the SDS-PAGE quantification method was an adequate HTP replacement for the more time consuming Bradford assay used for commercial and R&D batch quantification.

### HTPA

**Figure 5:**
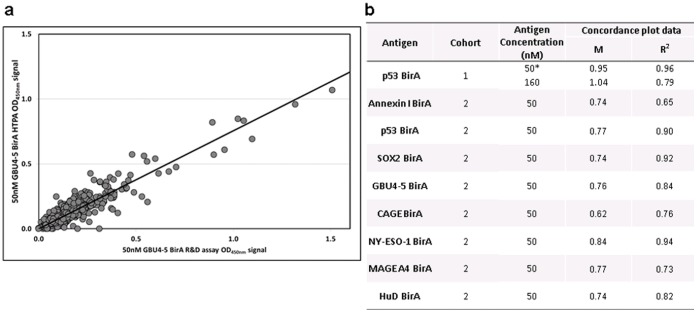
Assay format concordance. Commercial batches of several antigens were used for HTPA and R&D assay formats where AAbs in cohorts 1 or 2 serum samples were detected by both assay formats: a) an example of a concordance plot between HTPA and R&D assay OD data for 50 nM GBU4-5 BirA; b) the gradient (m) and fit (R2) values for the plot shown and the remaining data sets. *HTPA data was an average from 3 assay data sets, performed on different days.

**Figure 6:**
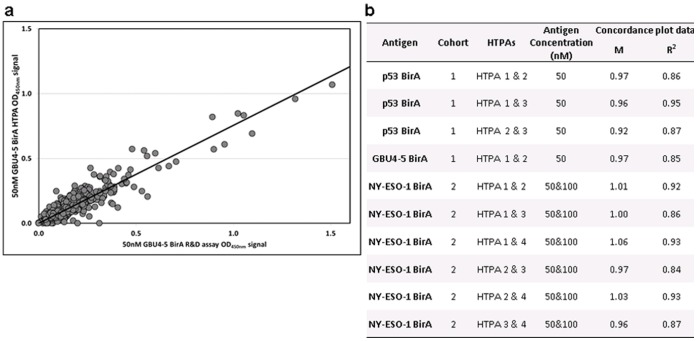
HTPA variability concordance. Commercial batches of several antigens were used to coat plates and AAbs in cohort 1 or 2 serum samples were detected by HTPA ELISA. Assays were repeated on different days to assess inter-assay variability: a) an example of a concordance plot between two identical HTP assay undertaken on different days; b) the gradient (m) and fit (R2) values for the plot shown and the remaining cohort 1 & 2 data.

The assay was developed using several lung panel antigens and two lung patient cohorts, used and described previously [6], [8]. The HTPA format was validated by comparison of commercial and R&D batches of *Early*CDT-Lung panel antigen performance by HTPA and R&D assay for two patient cohorts. Good correlation between the two formats was observed when comparing assay signal (Figure 5) indicating that the different plate layouts and protocols did not affect the assay output. Application of cut-offs also led to at least 95% agreement in the test result between HTPA and the R&D *Early*CDT assay for all the antigens investigated for both cohorts (Table 2). We also assessed assay variability by comparing HTPA signals for assays carried out on different days, where antigen plate coating solutions were also prepared independently (Figure 6). Excellent concordance was observed for all the antigens investigated for both cohorts.

**Table 2:** Summary of assay positivity and assay format agreement.

Antigen	Cohort	Assay format	Sensitivity (%)	Specificity (%)	Concordance (%)
**p53 BirA**	1	HTPA	34	100	100
		R&D *Early*CDT	34	100	
GBU4-5 BirA*	1	HTPA	13	94	96
		R&D *Early*CDT	9	100	
**p53 BirA**	2	HTPA	15	98	95
		R&D *Early*CDT	14	95	
**CAGE BirA**	2	HTPA	10	97	95
		R&D *Early*CDT	10	96	
**SOX2 BirA**	2	HTPA	4	100	99
		R&D *Early*CDT	3	100	
**NY-ESO-1**	2	HTPA	21	97	95
**BirA**		R&D *Early*CDT	22	97	
**GBU4-5 BirA**	2	HTPA	5	95	97
		R&D *Early*CDT	5	96	
**HuD BirA**	2	HTPA	4	98	98
		R&D *Early*CDT	4	98	
**MAGE A4**	2	HTPA	10	96	96
**BirA**		R&D *Early*CDT	10	95	

Comparison of HTPA and R&D *Early*CDT sample positivity results for commercial batches of certain lung panel antigens for Cohorts 1 and 2. * GBU4-5 compares an R&D batch (HTPA) to a commercial batch (R&D EarlyCDT), expressed under different conditions.

### HTPE Batch Scale up Reproducibility

**Figure 7:**
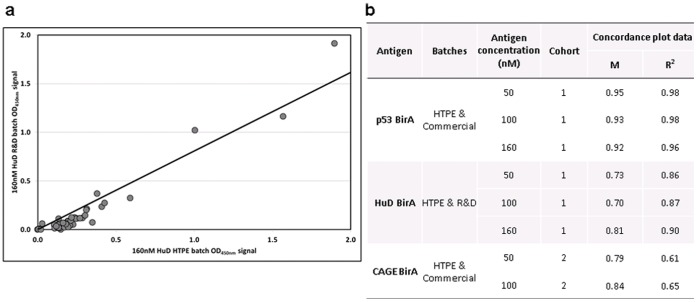
Batch concordance. HTPE, R&D and commercial batches of several antigens at 50, 100 and 160 nM were used to coat plates, and AAbs in cohort 1 (p53 BirA and HuD BirA) and 2 (CAGE BirA) serum samples were detected by HTPA: a) an example of a concordance plot between the HTPE and Commercial batch OD data for 160 nM HuD BirA; b) the gradient (m) and fit (R2) values for the plot shown and the remaining data sets. a) Concordance plot between the OD signal for all antigens at both concentrations; b) the gradient (m) and fit (R^2^) values for the plot shown with and without Mesothelin. The concordance for this antigen was poor, demonstrated by the fact that when the values are taken out a much greater concordance is observed for the remaining 5 antigens.

The HTPA performance of HTPE, R&D and commercial batches (Table 3) for some antigens was carried out to assess batch to batch reproducibility after scale up. Briefly, HTPE batches of some *Early*CDT-Lung panel antigens were produced under the same or similar expression conditions as R&D or commercial batches, and HTPA assay signal and result (either positive or negative) were compared for two patient cohorts (Figure 7). This not only assessed the effect on ELISA signal for the alternative scale, purification and storage of these different batch types, but also the alternative concentration determination (summarized in Table 1). As can be seen there is strong agreement of signal strength and also for resultant positivity and negativity between the two batches for several antigens (Figure 7). As we would expect the greatest concordance was seen for p53, which was the only antigen where truly equivalent HTPE and commercial batches (apart from the differences set out in Table 3) could be compared. The two p53 batches were expressed by the same cells, media and temperature and inclusion bodies were prepared and purified. The HuD and CAGE batches were expressed using different cells, media and temperature; inclusion bodies were prepared and purified for the HuD HTPE and CAGE batches, while full lysis was carried out for the HuD R&D batch.

**Table 3:** Summary of batch characteristics.

Batch type	HTPE	R&D	Commercial
**Scale (ml)**	3–30	200–500	5,000–20,000
**Cutlture Vessel**	Plate well	Shake flask	Fermenter
**Purification method**	IMAC*	IMAC	IMAC
**Refold method**	IMAC	Dialysis	Dialysis
**Storage buffer**	10% glycerol	0–50% glycerol	0–50% glycerol
**Quantification method**	SDS-PAGE	Bradford assay	Bradford assay

* IMAC = Immobilized metal chelate affinity chromatography.

**Table 4:** Comparison of characteristics for HTP and previous R&D strategies.

R&D Step	Characteristics	Previous strategy	HTP strategy
**Cloning**	**Cloning type:**	Restriction site	LIC
	**Number of constructs:**	2–3	1–96
	**Number of researchers:**	1	1
	**Timescale** * **(weeks):**	2–3	2–3
	**Cost per TAA ∼ (£):**	210	180
**Expression**	**Batch type:**	R&D	HTPE
	**Number of conditions:**	1∧	4“
	**Number of batches:**	7	192^†^
	**Number of researchers:**	1	1
	**Timescale’ (weeks):**	1	1
	**Cost per TAA ∼ (£):**	40	6
**Purification**	**Batch type:**	R&D	HTPE
	**Number of batches:**	4	192^†^
	**Number of researchers:**	2	1
	**Timescale (weeks):**	2	1
	**Cost per TAA ∼ (£):**	130	20
**Assay**	**Assay type:**	R&D 5 point assay	HTPA 2 point assay
	**Number of TAA tested:**	7	21
	**Number of researchers:**	4	4
	**Samples per day:**	200	200
	**Cost per TAA ∼ (£):**	108	36

* Experimental timescale. Clonal verification by sequencing results and analysis may take longer or require additional man power. ∼ Approximation of consumable costs.

∧ More flasks or different conditions may be required if yields are poor. “Over 90% of the constructs tested so far are expressed under the HTPE screen conditions. ^†^48 different proteins, 4 batches of each.‘Protein batches large enough for HTPA of up to 400 samples.

## Discussion

The two LIC sites of the vectors described here were designed to produce the desired fusion proteins with several differing considerations in mind. These included LIC cloning sequence requirements, incorporating flexible linkers between the antigen and tags and exposure of terminal antigen epitopes to maximize any binding interaction with AAb (Figure 1). It also enabled the production, by one person, of up to 96 constructs in parallel utilizing multi-well plate technologies with over 80% efficiency. HTP cloning not only allows the cloning of full length, but also multiple isoforms/variants or specific domains of any potential targets, identified from the literature. In this way the aim was to produce soluble and native epitope containing proteins which strongly bind any cancer associated serum AAb.

The HTPE screen was developed using *E.Coli*, but the methodology is also amenable to other expression systems, for example yeast, which we plan to investigate in the future. The authors recognize the limitation of bacterially expressed targets which are lacking in any potential glycan or conformational epitopes. Previous work investigating glycosylated and/or native TAAs utilizing mammalian, insect and yeast expression systems, as well as synthetic peptides, had however not identified any TAA superior to those produced in *E. Coli* with respect to distinguishing cancer patients from those with no malignant disease. With the added advantage of the large yields essential for a commercial ELISA test, it was felt that bacterial expression of potential targets was the most commercially and clinically viable method.

The HTPC and HTPE methodologies provide the opportunity to clone and express swiftly many genes and gene fragments to produce protein suitable for AAb assay formats. Screening these fusion proteins on a small scale is an attractive way of identifying constructs that are likely to be of use for identifying tumor associated AAb in patient sera, in a time and cost efficient manner. Any constructs identified in this way can then be scaled up and tested for reproducibility, evidence for which has been investigated previously [8]. The HTPE screen was successfully developed to meet our needs, providing mg quantities of many purified proteins, in a short timescale, for use in HTPA.

HTPE, R&D and commercial batches have the obvious disparity of scale but also culturing vessel and alternative methods of purification and storage (summarized in Table 3). During the development of the HTP expression and purification screen it was essential to determine whether the assay performance for HTPE proteins was likely to be a fair reflection of how these antigens would perform when scaled up to R&D or commercial batches. The agreement between assay results obtained using HTPE and R&D or commercial batch antigens (Figure 7) is impressive especially when considering the disparities for batch production, and in some cases expression conditions. These data suggest that HTP screening can identify strong lead candidates which are maintained through R&D to commercial batch scale-up; meeting the ultimate aim of the HTP discovery strategy set out in this body of work.

The combination of SDS-PAGE and anti-His control plate was deemed adequate for protein characterization, quantification and confirmation of plate coating accuracy and efficiency. The inclusion of the Tag only (BirA) and NY-ESO-1 control antigens in each HTPA also allowed assessment of SDS-PAGE quantification accuracy for every TAA HTPE batch assessed by HTPA, and also provided an internal control to determine assay variability (Figure 6, NY-ESO-1 data).

The *Early*CDT-Lung panel was developed by screening many antigens for AAb binding using the R&D 5 point assay. The five point assay is essential for accurate and reproducible classification of samples as positive or negative. However, in this format, only up to seven antigens can be screened per plate, hence large volumes of valuable, limited and often irreplaceable test sera are consumed in screening studies. This has proven to be an additional limitation to the costs and time needed to screen large amounts of antigens for their suitability for inclusion in AAb assay panels. To overcome this issue we developed a HTP, abridged, 2 point version (HTPA) of the 5 point assay. While some information is lost in the 2 point HTPA version it does allow the screening of 21 TAAs at a time. This HTPA assay, like the 5 point assay titration, utilizes one microtiter plate per serum sample requiring the same volume of serum per plate. We therefore can screen 21 antigens at the same time using the same amount of serum that previously screened only 7 TAAs for antigen discovery.

Any disparity in result (positive or negative) between the two assay formats (Table 2) was likely to be due to several predominantly unavoidable differences, summarized in Table 1. These discrepancies are acceptable when we consider the uses of the two assays. The HTPA is a discovery assay for quickly and economically identifying antigens worthy of further investigation for development or improvement of AAb panels, and as such does not replicate all the data obtained from the *Early*CDT assay. The *Early*CDT-Lung panel has been optimized for sensitivity and specificity for the detection of lung cancer, using several large multi-centre cohorts, optimizing batch protein production and developing a calibration system to reduce assay variability. Any lead antigens identified by HTPA would need to be validated by testing R&D and then commercial batches on the 5 point assay, in large case-control studies. It is therefore not essential for complete agreement between the two assay formats; rather an indication is required of how each TAA tested would be likely to perform in a clinical setting, both compared to and in combination with other AAb assays, to enable maximal utility and patient benefit.

To conclude, HTPC allows rapid cloning of many antigens, and/or fragments in parallel while optimizing epitope presentation thereby ensuring an improvement in turnover and efficiency compared to previous restriction site based cloning methods employed (Table 4).

HTPE allows the optimization of expression for these multiple constructs, in parallel, with the preparation of 48 different pure and fully characterized and quantified fusion proteins in a timescale of weeks (Table 4). Screening using the abridged HTPA format of the semi-automated AAb ELISA tests 21 antigens in the same time frame as the *Early*CDT assay format tests 7, using the same volume of serum by reducing the assay from 5 to 2 points (Table 4). This approach provides a quick and effective screening tool for new antigens, which are thought to perform well in AAb ELISA and are worthy of further investigation. The reproducibility following protein scale up demonstrated here ensures that confidence can be gained from initial HTP data. This strategy has already been used to identify new leads (which raise the sensitivity and/or specificity) for inclusion in the current *Early*CDT-Lung panel (Macdonald et al, manuscript in preparation).

By applying similar strategies to appropriate patient and high risk control populations the HTP approach will be used to develop AAb panels for other types of cancer (e.g. breast, colon, liver, ovary) allowing a more rapid assessment of newly identified biomarkers from the literature in a cost and time efficient manner (summarized in Table 4). Such technologies should ultimately lead to clinical tests being available to aid in the early detection of the cancers in a shorter time frame. This high throughput strategy also has the potential to be used as a research tool in the development of companion diagnostics for treatments based on using vaccines, monoclonal antibodies and signal transduction inhibitors for increased patient benefit. This biomarker screening strategy has been validated against a recognized standard (*Early*CDT-lung) which has successfully translated commercially to practical clinical utility. This is in stark contrast to previously published biomarker screening strategies and as such is distinctly significant.

## References

[1] Robertson JFR, Chapman C, Cheung K-L, Murray A, Pinder SE (2005). Autoantibodies in early breast cancer.. J Clin Oncol.

[2] Li YL, Karjalainen A, Koskinen H, Hemminki K, Vainio H (2005). p53 autoantibodies predict subsequent development of cancer.. Int J Cancer.

[3] Trivers GE, DeBenedetti VM, Cawley HL, Caron G, Harrington AM (1996). Anti p53 antibodies in sera from patients with chronic obstructive pulmonary disease can predate a diagnosis of cancer.. Clin Cancer Res.

[4] Chapman C, Murray A, Chakrabarti J, Thorpe A, Woolston C (2007). Autoantibodies in breast cancer: their use as an aid to early diagnosis.. Ann Oncol.

[5] Chapman C, Murray A, McElveen JE, Sahin U, Luxemburger U (2008). Autoantibodies in lung cancer: possibilities for early detection and subsequent cure; Thorax.

[6] Boyle P, Chapman CJ, Holdenrieder S, Murray A, Robertson C (2011). Clinical validation of an autoantibody test for lung cancer Ann Oncol.

[7] Desmetz C, Mange A, Maudelonde T, Solassol J (2011). Autoantibody signatures: progress and perspectives for early cancer detection.. J Cell Mol Med.

[8] Murray A, Chapman C, Healey G, Peek LJ, Parsons G (2010). Technical validation of an autoantibody test for lung cancer.. Ann Oncol.

[9] Lam S, Boyle P, Healey GF, Maddison P, Peek LJ (2011). EarlyCDT-Lung: An immunobiomarker test as an aid to early detection of lung cancer.. Cancer Prev Res.

[10] Chapman CJ, Healey GF, Murray A, Boyle P, Robertson C (2012). EarlyCDT®-Lung test: improved clinical utility through additional autoantibody assays. Tumor Biol 2012 Apr 11.. Epub ahead of print PMID 22492236.

[11] Structural Genomics Consortium, Architecture et Fonction des Macromolécules Biologiques, Berkeley Structural Genomics Center, China Structural Genomics Consortium, Integrated Center for Structure and Function Innovation, Israel Structural Proteomics Center, et al (2008). Protein production and purification.. Nature Methods.

[12] Aslanidis C, de Jong PJ (1990). Ligation-independent cloning of PCR products (LIC-PCR). Nucleic Acids Res..

[13] Parsy CB, Chapman CJ, Barnes AC, Robertson JF, Murray A (2007). Two step method to isolate target recombinant protein from co-purified bacterial contaminant SlyD after immobilised metal affinity chromatography.. J Chromatog B Anal Technol Biomed Life Sci.

[14] Howard PK, Shaw J, Otsuka AJ (1985). Nucleotide sequence of the birA gene encoding the biotin operon repressor and biotin holoenzyme synthetase functions of Escherichia coli.. Gene.

[15] Promega Corporation (2006). PinPoint™ Xa Protein Purification System Technical manual..

[16] Cronan JE (1990). Biotination of proteins in vivo:a posttranslational modification to label, purify and study proteins. J Biol. Chem..

